# Quantitative Lateral Flow Assay for Meropenem Determination: A Proof-of-Concept Study

**DOI:** 10.1021/acsomega.5c07602

**Published:** 2025-10-06

**Authors:** Vasin Vasikasin, Alaa Riezk, Richard C. Wilson, Timothy M. Rawson, Anthony E.G. Cass, Alison H. Holmes

**Affiliations:** † Centre for Antimicrobial Optimisation, 4615 Imperial College London, London SW7 2AZ, U.K.; ‡ Department of Internal Medicine, 37681 Phramongkutklao Hospital and Phramongkutklao College of Medicine, Bangkok 10400, Thailand; § David Price Evans Global Health and Infectious Diseases Research Group, University of Liverpool, Liverpool L69 7ZX, U.K.; ∥ Fleming Initiative, Fleming Centre, Imperial College London and Imperial College Healthcare NHS Trust, London SW7 2AZ U.K.

## Abstract

Therapeutic drug monitoring (TDM) is essential for optimizing antibiotic dosing, particularly in critically ill patients. However, conventional methods, such as LC/MS, have long turnaround times, limiting timely dose adjustment. We developed a novel competitive lateral flow assay (LFA) format with dual test lines previously validated for vancomycin and adapted it for Meropenem quantification. Using BlaR-CTD, a beta-lactam receptor protein, in place of an antibody and biotinylated BSA–Meropenem conjugated to gold nanoparticles, the LFA produced a concentration-dependent change in test line intensities. A custom image analysis algorithm showed strong correlation with Meropenem concentrations (*R* ^2^ = 0.9537). The platform demonstrates the potential for rapid, point-of-care antibiotic monitoring across diverse healthcare settings. While further optimization is needed for low-concentration accuracy, this proof-of-concept supports broader applicability to other beta-lactams.

## Graphical abstract

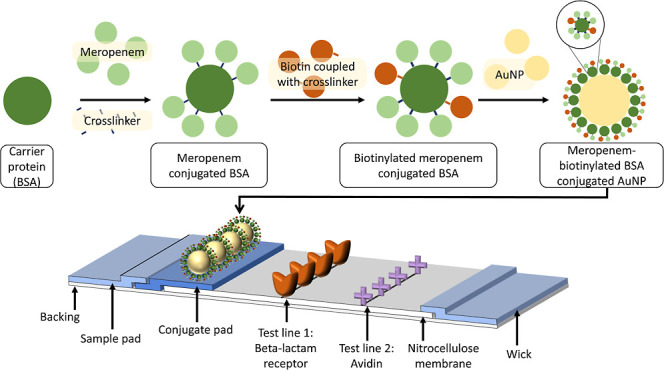

## Introduction

A key strategy in addressing antimicrobial resistance is the optimization of antibiotic dosing through therapeutic drug monitoring (TDM), which involves measuring plasma antibiotic concentrations to guide dose adjustments.^1^ In critically ill patients, TDM for beta-lactams—such as Meropenem—is recommended due to its demonstrated benefits in achieving therapeutic drug concentrations.^2^ However, the prolonged turnaround time associated with conventional TDM methods often limits timely dose modifications, reducing their clinical impact.^3^

Liquid chromatography–mass spectrometry (LC/MS) is the most widely used method for beta-lactam TDM.^4,5^ Although LC/MS offers relatively short analytical run times (3–30 min),^6^ the overall turnaround time typically ranges from 18 to 24 h due to sample processing and laboratory workflows.^3^ In real-world settings, delays can extend up to 4 days,^7^ significantly hindering timely therapeutic interventions, especially in critically ill patients.

We previously developed a novel competitive lateral flow assay (LFA) format for vancomycin, featuring two test lines: an antibody line and an avidin line.^8^ This design utilizes the biotin–avidin interaction to capture excess conjugate, allowing for differential signal analysis between the two lines. The drug concentration can then be extrapolated based on the signal intensities with high accuracy.

In this study, we adapted and expanded this LFA format for Meropenem quantification to demonstrate the platform’s potential to quantify other antibiotic classes. This proof-of-concept study focuses on analytical feasibility while recognizing that full bioanalytical validation and clinical testing remain necessary for clinical use.

## Material and Methods

The methodology followed the development framework previously used for the vancomycin LFA.^8^ Chemicals, reagents, and the gold nanoparticle (AuNP) conjugation method, image interpretation, and curve fitting model were similar, with some adjustments made to reagent concentrations for optimal performance, as detailed below.

Due to the lack of a high-specificity antibody for beta-lactams, the antibody component was replaced with the carboxy-terminal domain of the sensor-transducer beta-lactam receptor (BlaR-CTD) derived from *Bacillus licheniformis*.^9^ The BlaR-CTD gene was cloned, expressed, purified, and concentrated to 2 mg/mL, as detailed in Supporting Information Appendix 1.

Meropenem was conjugated to 2-Mercaptoethanol activated bovine serum albumin (BSA) using EDC, followed by biotinylation. For AuNP labeling, the biotinylated BSA–Meropenem conjugate was mixed with AuNPs and stabilized with BSA and polyethylene glycol (PEG).

The LFA strips were prepared with two test lines on nitrocellulose: TL1 (drug-binding BlaR-CTD line) and TL2 (capture avidin line). Strips were cut to a 3.175 mm width, and 30 μL of the sample was used per test. The run buffer contained phosphate-buffered saline (PBS) with Tween, BSA, PEG, and sucrose.

## Results

The LFA produced a clear and progressive variation in signal intensity at both test lines, corresponding to different Meropenem concentrations. Image analysis using a custom algorithm generated a dose–response model defined by the equation: *y* = 1.7182 × *x* ^0.1451^, where *y* represents Meropenem concentration (μg/mL) and *x* is the signal ratio from the two test lines (TL2/TL1). The model demonstrated a strong correlation with an *R* ^2^ value of 0.9537, as illustrated in Figure 1.

**Figure 1:**
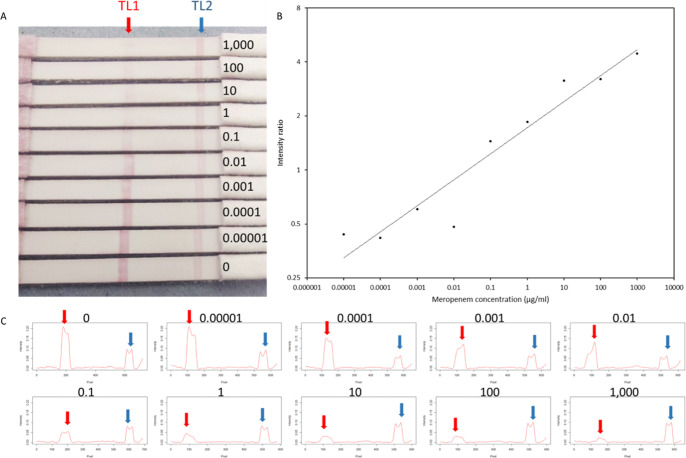
Analytical performance of the novel competitive LFA for Meropenem. (A) Visual appearance of LFA strips tested with varying concentrations of Meropenem. (B) Calibration curve of the assay, demonstrating the quantitative relationship between signal intensity and Meropenem concentration. (C) Signal intensity profiles corresponding to each strip, illustrating the relative responses at different concentrations. Numerical values indicate Meropenem concentrations (μg/mL). Red arrows denote test line 1 (TL1), and blue arrows denote test line 2 (TL2).

## Discussion

This proof-of-concept study demonstrates that the novel competitive LFA format holds promise for the quantification of Meropenem and potentially other beta-lactam antibiotics.

The development of the Meropenem assay serves as a proof of concept, supporting the adaptability of this LFA platform to other antibiotic classes. Visual inspection of the LFA strips revealed a consistent trend: increasing Meropenem concentrations reduce TL1 intensity and relatively increase TL2 intensity. Notably, at zero Meropenem concentration, the second line remained faintly visible, suggesting that the biotinylation protocol may require further optimization to improve baseline signal clarity.

The platform’s rapid turnaround (<30 min) compares favorably with LC/MS, which typically requires 18–96 h, and could enable more timely dose adjustments in clinical practice.

However, several important limitations must be acknowledged. This work did not include full bioanalytical method validation in accordance with FDA/EMA guidelines, including assessments of accuracy, precision, specificity, recovery, inter- and intraday reproducibility, or limit of detection/quantification. Furthermore, clinical applicability was not evaluated, as no serum or plasma samples from patients were tested. Head-to-head comparisons with LCMS are necessary to establish the concordance and reliability for TDM.

Despite this limitation, a concentration-dependent increase in the ratio between the two test lines was observed, supporting the assay’s validity. However, the greatest deviation between the predicted and actual concentration occurred at 0.01 μg/mL. This discrepancy may stem from physical irregularities in the LFA strips—such as variations in line width or membrane quality—or from limitations in the image analysis algorithm and curve-fitting model.

This study demonstrates the feasibility of adapting a dual-line competitive LFA platform for Meropenem quantification. While the assay shows promising alignment with therapeutic ranges and analytical correlation, it remains an early proof of concept. Full bioanalytical validation and clinical testing are required before the assay can be considered for monitoring Meropenem concentrations during routine clinical practice.

## Supplementary Material



## Notes

Expression and characterization of BlaR-CTD (PDF)
