# Supplementary material for: Quantitative Lateral Flow Assay for Meropenem Determination: A Proof-of-Concept Study
Source: ACS Omega. 2025 Oct 6;10(40):47566–9. doi: 10.1021/acsomega.5c07602 (PMC12529179; doi:10.1021/acsomega.5c07602)
Supplement: Supplementary file 1 [file ao5c07602_si_001.pdf]

Quantitative Lateral Flow Assay for Meropenem Determination: a Proof-of-Concept Study

Vasin Vasikasin[1,2], Alaa Riezk[1], Richard C Wilson[1,3], Timothy M Rawson[1,3,4*], Anthony EG Cass[1], Alison H Holmes[1,3,4]

[1] Centre for Antimicrobial Optimisation, Imperial College London, UK.

[2] Department of Internal Medicine, Phramongkutklao Hospital and Phramongkutklao College of Medicine, Bangkok, Thailand

[3] David Price Evans Global Health and Infectious Diseases Research Group, University of Liverpool, Liverpool, United Kingdom

[4] Fleming Initiative, Fleming Centre, Imperial College London and Imperial College Healthcare NHS Trust, United Kingdom

* corresponding author

Corresponding author

Timothy Miles Rawson

Centre for Antimicrobial Optimisation, Imperial College London, UK

Commonwealth Building, Hammersmith Hospital Campus, Du Cane Road, London, W12 0NN

timothy.rawson07@imperial.ac.uk

Supplementary Appendix

Expression and characterisation of BlaR-CTD

The process for expression and characterisation of BlaR-CTD involves extraction of gene of interest, annealing of the gene, plasmid propagation, and protein expression and purification, as summarised in Figure S1.

Figure S1 Steps involved in the expression of BlaR-CTD

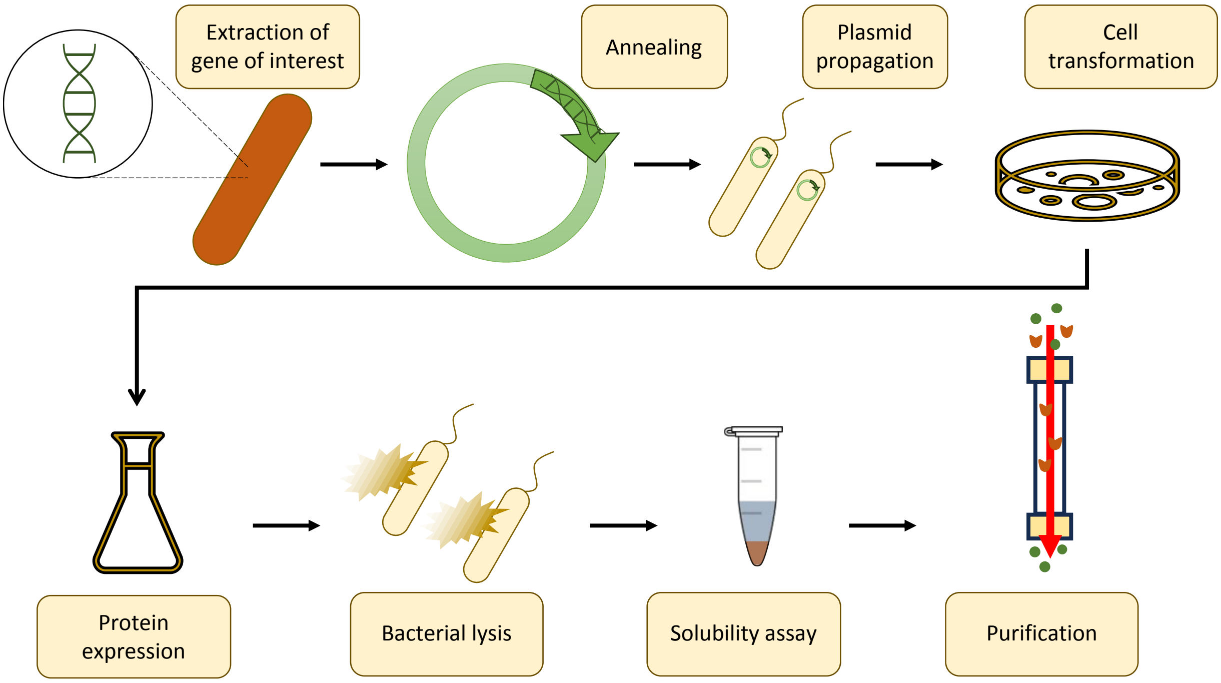

Extraction of gene of interest

Freeze-dried *Bacillus licheniformis* from NCTC was diluted with Super Optimal broth with Catabolite repression (SOC) media for 15 minutes and culture overnight on sheep blood agar at 37°C. Genomic DNA was isolated from the supernatant by heat treatment of the overnight colony in sterile water at 95°C for 15 minutes and centrifuged for 5 minutes. PCR of BLAR-CTD gene from the genomic DNA was performed using two different proofreading DNA polymerase enzyme master mixes,

KOD Hot Start and Platinum SuperFi II Green, and the BLAR-CTD primer set described in Table S1. DNA gel electrophoresis was used to confirm the PCR product size. Then, PCR product was purified using QIAquick PCR Purification kit. DNA concentration was then measured along with A260/A280 and A260/A230 ratios to determine the purity.

Annealing of BLAR-CTD gene to plasmid

Linearisation of pET-YSBLIC3C plasmid was done using KOD Hot Start master mix and the pET-YSBLIC3C plasmid primer set described in Table S1. The product was treated with DpnI enzyme and incubated at 37°C for 1 hour to degrade the template DNA.

Table S1 The sequences of primers used and the expected size of PCR products

Primers	Sequences	Gene	Expected size (bp)
pET-YSBLIC3C Fwd	5’-CGCGCCTTCTCCTCACATATGGCTAGC-3’	pET-YSBLIC3C plasmid	5,419
pET-YSBLIC3C Rev	5’-**T**TGCTGGTCCCTGGAACAGAACTTCC-3’		
BLAR-CTD Fwd	5’-CCAGGGACCAGCA**ATG**CAAAGAGATACGCACTT-3’	BLAR-CTD (BlaR-CTD)	795
BLAR-CTD Rev	5’-GAGGAGAAGGCGCGTTA**TTA**TCGGGAAGCGGATGG-3’		
T7 Fwd	5’-TAATACGACTCACTATAG-3’	BLAR-CTD with T7 promoter	1,111
T7 Rev	5’-GCTAGTTATTGCTCAGCGG-3’		

Fwd: forward primer, Rev: reverse primer, A: adenine nucleotide, T: thymine nucleotide, C: cytosine nucleotide, G: guanine nucleotide, green nucleotides: starting codons and following sequence, red nucleotides: stop codon and previous sequence, yellow and blue nucleotides: complementary DNA nucleotide bases which were expected to be annealed.

To excise 3’-5’ and create overhangs for annealing between plasmid vector and gene insert, T4 DNA polymerase enzyme, T4 DNA polymerase buffer, and DTT were mixed. dTTP was used for creating overhangs for plasmid vector, whereas dATP was used

for gene insertion. The reaction was created by incubation at 22°C for 30 minutes and stopped by incubation at 75°C for 20 minutes.

The gene insert was annealed into the plasmid vector by incubating at 22°C for 10 minutes. Then the product was mixed with EDTA and incubated at 22°C for another 10 minutes. DNA gel electrophoresis was used to confirm the annealing as compared to negative control using plasmid vector.

Transformation for plasmid propagation

The recombinant plasmid was transformed into OneShot Top 10 chemically-competent *E.coli* by incubating on ice for 30 minutes, heat shock at 42°C for 30 seconds, and incubating in SOC medium at 37°C for 1 hour with shaking at 225 rpm. The bacteria with recombinant plasmid and without recombinant plasmid (negative control) were spread on LB agar containing kanamycin 30 ug/mL and were incubated overnight at 37°C. The numbers of colonies were compared between the bacteria with recombinant plasmid and the negative control.

Seven discrete colonies and one colony of negative control were picked for PCR confirmation using T7 primer set described in Table S1. A subset of colonies with confirmation by PCR were cultured overnight in LB broth containing kanamycin. The plasmids were isolated with QIAprep Spin Miniprep Kit and sent for Sanger sequencing.

Transformation for protein expression

After the sequence confirmation, the isolated plasmids were transformed into BL21(DE3) competent *E.coli* by incubating on ice for 30 minutes, heat shock at 42°C for 30 seconds, and incubating in SOC medium at 37°C for 1 hour with shaking at 225

rpm. The empty plasmid was also transformed as a negative control. The bacteria were sub-cultured into LB broth supplemented with kanamycin for overnight culture at 37°C.

The overnight cultures were sub-cultured into LB broth supplemented with kanamycin and grew at 37°C until reaching log phase, at OD_{600} of 0.4-0.8. To induce protein expression, the cultures were mixed with 0.2mM isopropyl β-D-1-thiogalactopyranoside (IPTG) and incubated overnight at 24°C with shaking. The overnight cultures were pelleted and resuspended in BugBuster solution supplemented with lysozyme and benzonase nuclease. The solutions were then centrifuged. The supernatants were transferred for soluble fraction analysis. The pallets were resuspended in lysis buffer and transferred for insoluble analysis.

Sodium dodecyl sulfate–polyacrylamide gel electrophoresis (SDS-PAGE) of the overnight cultures before IPTG induction, the cultures in log phase before IPTG induction, the cultures after IPTG induction, the soluble solution, and insoluble solution of both bacteria with recombinant plasmid and empty plasmid was done. Western blot of the SDS-PAGE with anti-His antibody was used to confirm the expression of his-tag.

Protein expression and protein purification

0.2 mM IPTH induction with overnight incubation at 25°C. The his-tag soluble BLAR-CTD was purified with two different methods using gravity column for comparison, nickel-ion nitrilotriacetic acid (Ni-NTA) resin and cobalt-ion (Co) resin.

For Ni-NTA resin, two sets of buffers were used. The first set consisted of 300 mM NaCl, 50 mM NaH_2PO_4 (pH 8.0) in water (Ni-NTA Buffer Kit) with 10 mM imidazole,

20 mM imidazole, and 250 mM imidazole as binding, washing, and eluting buffer, respectively. The second set consisted of 500 mM NaCl, and 20 mM Tris-HCl (pH 7.9) in water (His-Bind Buffer Kit) with 5 mM imidazole, 60 mM imidazole, and 1 M imidazole as binding, washing, and eluting buffer, respectively. Additionally, different imidazole concentrations were added in washing step to the final concentration ranging from 20 to 120 mM of imidazole.

For the TALON resin, the buffer consisted of 500 mM NaCl, 20 mM Na_3PO_4 (pH 7.4) in water with imidazole ranging from 0 to 500 mM were tried to find the optimal concentration for binding, washing, and eluting buffer.

Regarding the volume, each 18-20 mL of cell lysate obtained from 300 mL of culture broth can be loaded to a 1-mL resin column. For each 1 mL resin, 10 mL, 20 mL, and 4 mL were used for binding buffer, washing buffer, and eluting buffer, respectively.

The purified product was dialysed in PBS with a 12kDa dialysis kit to remove imidazole. For 8-10 mL of the purified product, 800 mL PBS was used and exchanged twice a day for three days. The product was then transferred to a centrifugal filter to concentrate and remove low molecular weight protein contaminant. For 8-10 mL of dialysed protein, 4,000 g for 12 minutes was used to obtain a 1 mL of concentrated protein.

Bicinchoninic acid assay (BCA) assay was used for protein quantification. The concentrated protein was used as undiluted, together with two-, and four-time dilution to be compared with BSA at a concentration of 0 to 2 mg/ml. The calibration curve was developed using quartic polynomial ($y = ax^4 + bx^3 + cx^2 + dx + e$) formula. The mean value from all the values obtained was used. BLAR-CTD was diluted and kept at 2 mg/mL at -20 °C.

BlaR-CTD gene (Figure S2)

The nucleotide sequence (765 bp) and corresponding translated amino acid sequence. The arrow depicts the direction of transcription. The image was generated with Benchling [Biology Software].

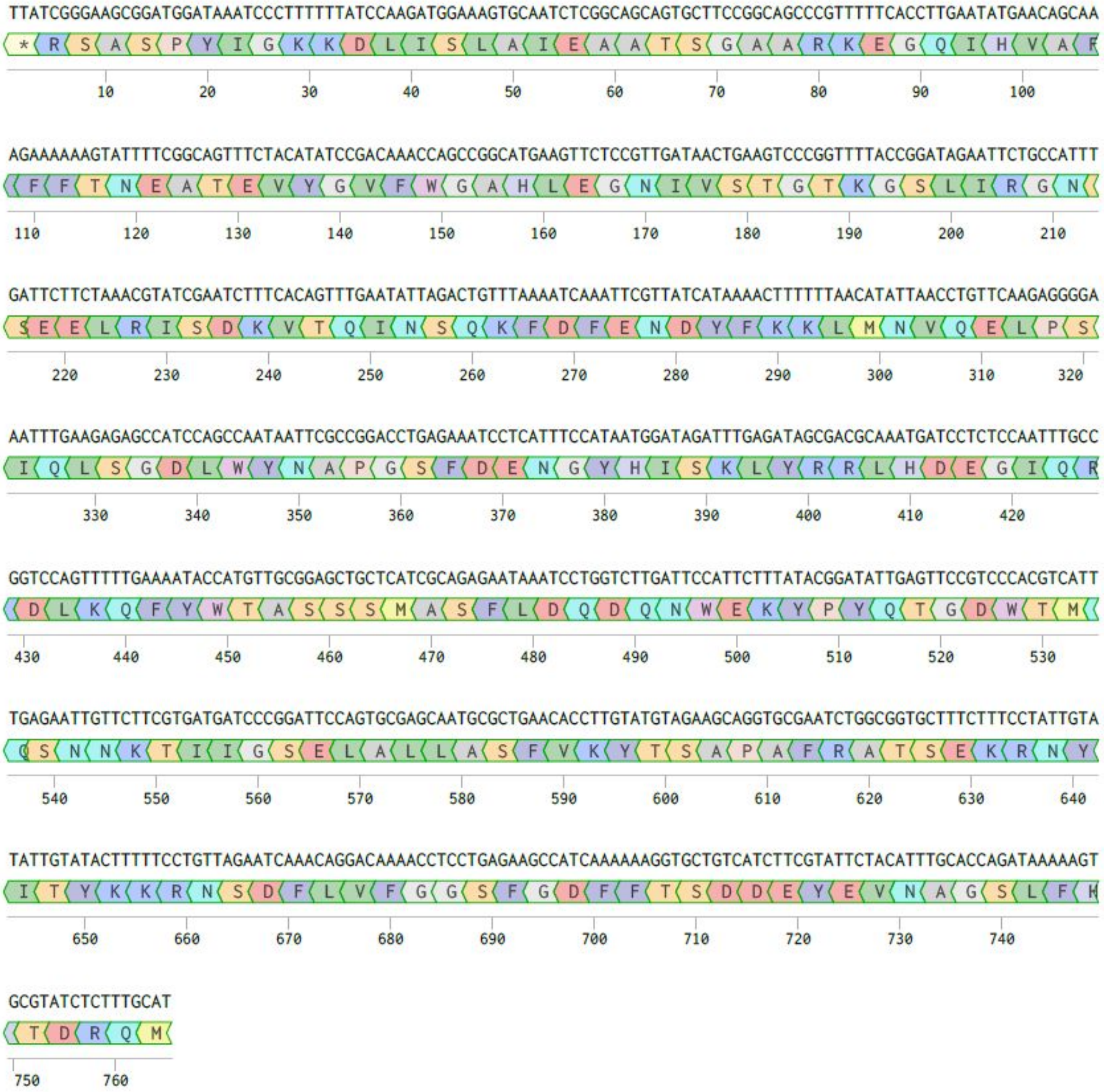

Figure S2 BlaR-CTD gene

BlaR-CTD inserted pET-YSBLIC-3C plasmid map (Figure S3)

The plasmid map featuring the insertion of the BlaR-CTD gene, along with its associated genes, was created using Benchling [biology software]. The plasmid measures 6,161 base pairs in size. YSBL3C_F and YSBL3C_R denote the forward and reverse primers employed for annealing, as detailed in Table S1.

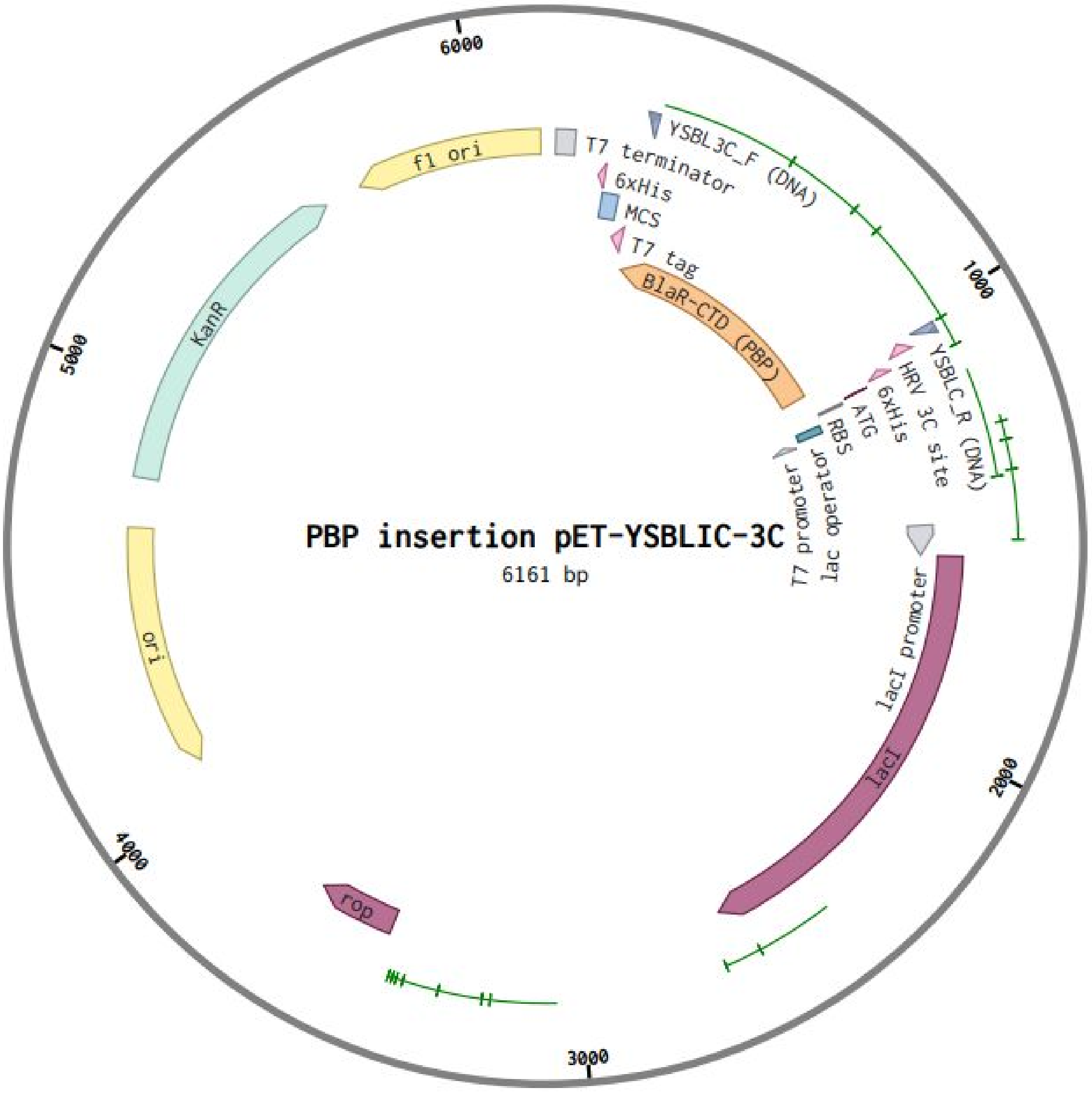

Figure S3 BlaR-CTD inserted pET-YSBLIC-3C plasmid map
